# Nanoparticle pre-treatment for enhancing the survival and activation of pulmonary macrophage transplant

**DOI:** 10.1007/s13346-023-01319-6

**Published:** 2023-03-14

**Authors:** Bader M. Jarai, Kartik Bomb, Catherine A. Fromen

**Affiliations:** Department of Chemical and Biomolecular Engineering, University of Delaware, 150 Academy St., Newark, DE 19716 USA

**Keywords:** Macrophages, Survival, Cell therapy, Nanoparticles, Polarization, Pulmonary transplant

## Abstract

Despite recent clinical successes of chimeric antigen receptor T cell therapies in treating liquid cancers, many lingering challenges stand in the way of therapeutic translation to broader types of malignancies. Macrophages have been proposed as alternatives to T cells given macrophages’ advantages in promoting tumor infiltration, acquiring diverse antigens, and possessing the ability to continuously stimulate adaptive responses. However, the poor survival of macrophages upon transplantation in addition to transient anti-tumor phenotypical states have been major obstacles standing in the way of macrophage-based cell therapies. Given recent discoveries of nanoparticle strategies in improving macrophage survival and promoting phenotype retention, we herein report the ability to extend the survival and phenotype of macrophage transplants in murine lungs via pre-treatment with nanoparticles of varying degradation rates. Macrophages pre-treated with 100 µg/ml dose of poly(ethylene glycol) diacrylate nanoparticle formulations improve pulmonary macrophage transplant survival over untreated cells beyond 7 days, where degradable nanoparticle formulations result in over a 50% increase in retention of transplanted cell counts relative to untreated cells. Furthermore, pre-treated macrophages more efficiently retain an imposed pro-inflammatory-like polarization state following transplantation out to 7 days compared to macrophages pre-treated with a classical pro-inflammatory stimulus, interferon-gamma, where CD86 costimulatory molecule expression is greater than 150% higher in pre-treated macrophage transplants compared to untreated counterparts. These findings provide an avenue for a major improvement in the lifespan and efficacy of macrophage-based cell therapies and have broader implications to other phagocyte-based cellular therapeutics and administration routes.

**Graphical Abstract:**

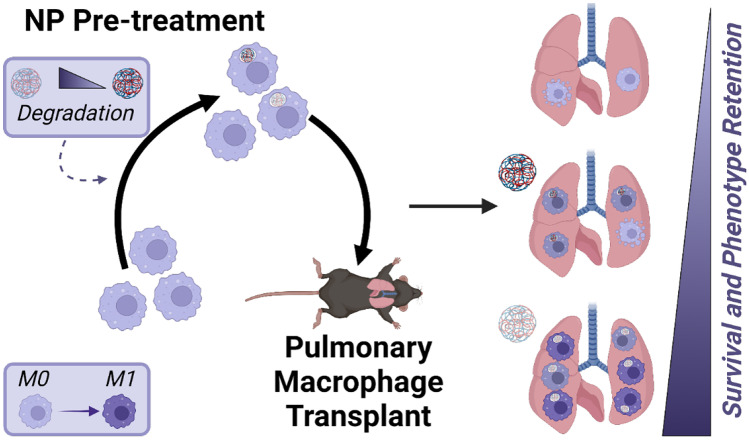

**Supplementary Information:**

The online version contains supplementary material available at 10.1007/s13346-023-01319-6.

## Introduction

Cell therapies have emerged as a paradigm shift in the therapeutic space, especially in treating neurodegenerative diseases [1], autoimmune disorders [2], and cancers [3]. Most notably, chimeric antigen receptor (CAR) T cell therapy has shown clinical success in treating many blood cancers [4]. However, CAR T cell therapies are limited by several roadblocks, including low efficacy against solid tumors, poor persistence ex vivo and in vivo, rapid loss of target antigen, and severe inflammatory side effects and toxicities [5]. Furthermore, CAR T therapies require engineering to include a specific target antigen, which limits therapeutic function to the specified target, highlighting a shortcoming and potential antigenic mismatch in heterogeneous and/or rapidly mutating cancers.

Accordingly, macrophages have emerged as potential cell therapy candidates, providing advantages over CAR T cell therapy owing to their ability to infiltrate strong immunosuppressive environments of solid tumors, their constant surveillance of tumor antigens, and their capacity for in situ education of lymphocytes [6]. Macrophages belong to a class of innate antigen-presenting cells (APCs), which are terminally differentiated cells that plastically respond to their microenvironment and take on activated phenotypes that can modulate the adaptive immune system towards antigen-specific anti-tumor function. Through incorporation of antigen-specific CAR domains, CAR M therapy has been shown to shift the tumor microenvironment towards the pro-inflammatory state desired for effective tumor therapy [7], enhance macrophage phagocytosis [8, 9], and achieve high cell expansion and transcription efficiency from inducible pluripotent stem cells [10]. These seminal works have resulted in the first Phase 1 clinical trial with CAR M therapies and second-generation CAR M therapies are in ongoing development [6, 9]. While CAR M therapy holds potential for the development of many novel therapeutics, the nascent field has met crucial roadblocks, such as macrophage anti-cancer capabilities, cell expansion and survival, and phenotype plasticity [9]. In the historical understanding of macrophage polarization, macrophage phenotype can be described by two extremes: classically activated, pro-inflammatory, anti-tumor M1 macrophages and alternatively activated, wound-healing, tumor-supporting M2 macrophages [11–13], with resident tumor-associated macrophages (TAMs) comprising a specialized M2-like subset and key regulator of the tumor immune microenvironment [14]. Cell plasticity between these phenotypes contributes to decreased therapeutic efficiency, as CAR M cells may rapidly lose the desired anti-cancer M1 phenotype in immunosuppressive tumor environments, resulting in reduced transplant viability, poor antigen presentation, and weak T cell activation and anti-tumor responses. Therefore, methods to improve sustained phenotypical responses (ideally M1-like) and high transplant viability are needed to realize a macrophage-based platform for cell therapies.

We have previously shown that nanoparticle (NP) internalization promotes the survival of ex vivo primary macrophages with implications for parallel NP-induced survival effects for macrophages in vivo [15]. Furthermore, we have demonstrated that NP formulation and degradation rate is an opportune parameter to modulate ex vivo primary macrophage survival and activation, where rapidly degrading pro-M1 formulations caused significant enhancement to macrophage survival and expression of M1-like markers [16]. Herein, we present a novel strategy to utilize previously discovered NP-induced macrophage longevity to enhance the survival of pulmonary macrophage transplant (PMT) and drive M1-like phenotype retention. We demonstrate that bone marrow–derived macrophages (BMMs) pre-treated with degradable pro-M1 NP formulations show enhanced survival in a murine model of PMT compared to untreated transplant cells. The pulmonary route is investigated for two key reasons: (1) PMT has been evaluated clinically in humans for pulmonary alveolar proteinosis (hPAP), where PMT has outstanding translational potential for treating certain airway conditions [17], and (2) delivering directly to the lung compartment, where minimal cell trafficking away from the lung is expected, allows us to perform a more restricted tissue analysis to accurately quantify the small population of surviving, non-proliferative transplanted cells. Compared with systemically administered treatments, direct delivery to the lung has the potential to limit off-target side effects and provide superior localized responses in treating pulmonary-related illnesses, where cell therapies remain an untapped opportunity in this regard. Poly(ethylene glycol) diacrylate (PEGDA) NP formulations containing 0% and 20% of degradable HS-PEG-SH linker cause a 31% and a 54% increase in transplant survival compared to untreated transplants 3 days post administration, with a retention of transplant survival over 7 days, especially in rapidly degradable 20% HS-PEG-SH formulations. Furthermore, NP-treated transplants show improved retention of M1-like phenotype compared to untreated transplant and even cells pre-treated with interferon-gamma (IFN-γ), a potent M1-stimulating cytokine. Notably, CD86 costimulatory molecule expression for 0% and 20% NP-treated transplants are 153% and 165% higher than that of untreated transplant, respectively, over 7 days, exceeding IFN-γ-treated transplants, which show CD86 levels that are indistinguishable from untreated counterparts. Thus, NP composition and degradation rate impacts the survival and phenotype of the transplanted macrophages, with rapidly degrading, M1-inducing formulations showing improvements over slowly degrading formulations. These findings provide a proof-of-concept utility of NPs for improving macrophage transplant survival and have implications in PMT and macrophage-based cell therapies broadly, especially for thoracic malignancies and immune disorders.

## Materials and methods

### Nanoparticle synthesis

50wt% PEGDA-based hydrogel NPs were prepared as described previously [15]. Briefly, to generate 0% HS-PEG-SH and 20% HS-PEG-SH PEGDA NPs (denoted as 0% and 20%, respectively), monomer molar compositions (Table 1) were prepared by varying mol% of poly(ethylene glycol) diacrylate (PEGDA) M_n_ = 700 (Millipore Sigma), thiol-PEG-thiol (HS-PEG-SH) M_n_ = 600 (Creative PEGWorks), 1,6-hexanediol dimethacrylate (HDDMA) (Millipore Sigma), and 2-carboxyethyl acrylate (CEA) (Millipore Sigma). Monomer mixture was combined with diphenyl(2,4,6-trimethylbenzoyl) phosphine oxide photoinitiator (PI) (Millipore Sigma) and fluorescent label cyanine 5 (Cy5) maleimide (AAT Bioquest) (1 mg and 0.05 mg, respectively). The resulting pre-particle formulations were combined with methanol (Fisher Scientific) at a ratio of 1:1 by mass. Miniemulsions were formed by emulsifying 100 μl of the mixture with 1 ml of silicone oil AP1000 (Millipore Sigma) via vortex mixing and sonication. The emulsion was then exposed to UV light (APM LED UV Cube, 365 nm wavelength, ∼28 cm from the light source, ∼5–10 mW/cm^2^) for 46 and 56 s for 0% and 20% NP formulations, respectively. The resulting suspensions were washed with 1 ml of n-hexanes (Millipore Sigma) followed by two more washes with 1 ml of 200 proof ethanol.

**Table 1:** Molar compositions of monomer mixtures (mol%) of NPs used in transplant studies

**NP**	**Formulation**	**PEGDA**	**HS-PEG-SH**	**HDDMA**	**CEA**
**0%**	0% HS-PEG-SH	75	0	5	20
**20%**	20% HS-PEG-SH	55	20	5	20

### Dynamic light scattering (DLS) and zeta potential

Hydrodynamic diameters and polydispersity indices (PDIs) of the 0% and 20% NP formulations were measured via DLS using a Malvern Zetasizer Nano ZS. Zero percent and 20% NP samples were prepared by adjusting sample concentrations to ∼0.1 mg/ml in water. Hydrodynamic diameters and PDIs were assessed from at least three measurements. NP samples were prepared for zeta potential measurement by diluting in 0.1 × PBS. Zeta potentials were measured from three independently synthesized samples.

### Thermal gravimetric analysis (TGA)

NP concentrations for dosing were determined via thermal gravimetric analysis (TGA) using TA Instruments TGA 550. After the final ethanol wash, 50 μl of NP suspensions was transferred to sample pans in triplicates. A temperature ramp to 120 °C followed by a 30-min isothermal step was carried out to ensure ethanol evaporation and the remaining mass of the NPs in the 50 µl suspension was determined via a mass reading at the end of the isothermal step. The same protocol was repeated after washing NPs in water before finally resuspension in DMEM/F-12 media containing 10% fetal bovine serum (FBS) and 1% Penicillin–Streptomycin to ensure accurate NP dosing.

### Animals

All studies involving animals were performed in accordance with National Institutes of Health guidelines for the care and use of laboratory animals and approved by the Institutional Animal Care and Use Committee (IACUC) at the University of Delaware. All institutional and national guidelines for the care and use of laboratory animals were followed. C57BL/6 J and B6.SJL-*Ptprc*^*a*^* Pepc*^*b*^/BoyJ mice (Jackson Laboratories) were housed in a pathogen-free facility at the University of Delaware, given free access to water and chow, and maintained under a normal daily light cycle. Female B6.SJL-*Ptprc*^*a*^* Pepc*^*b*^/BoyJ mice 6 to 10 weeks of age were used to obtain BMMs for transplant owing to their distinctive CD45.1 alloantigen for ease of flow cytometric identification and tracking [18]. Female C57BL/6 J mice 6 to 10 weeks of age were used as transplant hosts.

### Primary cell isolation and differentiation

BMMs were isolated from mice according to standard protocols [19]. Briefly, bone marrow was isolated from femurs and tibias of female B6.SJL-*Ptprc*^*a*^* Pepc*^*b*^/BoyJ mice and cells were seeded in eight T-75 cell culture flasks and cultured in the presence of DMEM/F-12 media (Corning) containing 20% FBS, 30% L929 cell conditioned media, and 1% Penicillin–Streptomycin (BMM differentiation media). Three days following seeding, an equal volume of BMM differentiation media was added to the flasks. BMM differentiation media was removed on day 7 and cells were cultivated by scraping and used for experiments in DMEM/F-12 media containing 10% FBS and 1% Penicillin–Streptomycin. Confirmation of macrophage phenotype was performed prior to transplant, as described in the following sections.

### Pulmonary macrophage transplant

In preparation for macrophage transplant, mice received three daily doses of clodronate liposomes (50 µl per dose, 5 mg/ml clodronate) (Liposoma BV) to deplete the resident macrophage population [20] and macrophage transplant was carried out 2 days following the third dose of clodronate liposomes. After differentiation, BMMs were plated in T-75 cell culture flasks (1.5 × 10^7^ cells per flask) and allowed to adhere overnight prior to NP treatment. BMMs were then dosed with 100 µg/ml Cy5-labelled NPs resuspended in DMEM/F-12 media containing 10% FBS and 1% Penicillin–Streptomycin. At 24 h following NP treatment, cells were washed twice with PBS and detached using scraping. Cells were suspended by gentle pipetting and counted using Countess II Automated Cell Counter (Thermo Fisher) following staining with trypan blue dye (Gibco) to exclude dead cells from counts. Live cell concentration was adjusted to 4.0 × 10^7^ cells/ml for all conditions in preparation for transplants. Cells were transplanted in mice via orotracheal instillation (50 µl) of cell suspension [21, 22].

### Transplant survival assessment

At analysis endpoints, mice were euthanized via CO_2_ overdose and lungs were extracted and digested with 5 mg/ml type IV collagenase (Gibco) in PBS supplemented with 2% FBS for 2 h at 37 °C, along with physical agitation. Digested lungs were suspended by gentle pipetting and passed through a 70-µm strainer and spun down at 500 RCF for 5 min. Lung digests were then resuspended in red blood cell lysis buffer (Invitrogen) for 60 s before quenching with PBS supplemented with 2% FBS. Digests were then washed twice with PBS supplemented with 2% FBS and Lymphoprep™ density gradient (STEMCELL Technologies) was used to isolate mononuclear cells for analysis. Briefly, a 1:1 ratio of cell suspension to density gradient was used and spun down using a precooled centrifuge at 800 RCF for 20 min without the brake. The buffy coat was then isolated. Cells were washed twice with PBS and then stained with Zombie Yellow™ Fixable Viability Kit (Biolegend) according to manufacturer’s guidelines for flow cytometric assessment of cell viability. Cells were then incubated with anti-CD16/32 (Biolegend) for 10 min to block Fc receptors and then stained with CD45.1-Pacific Blue to identify transplant cells. Cells were then analyzed using ACEA NovoCyte Flow Cytometer. Transplant survival was assessed by determining the counts and percentages of CD45.1 + /Zombie Yellow– populations.

### Transplant phenotype assessment

Similar to sample preparation for transplant survival assessment, pre-transplant and post-transplant (following density gradient preparation) cells were blocked with anti-CD16/32 for 10 min and then stained with CD86-AlexaFluor700, and I-A/I-E-Brilliant Violet 785™ antibodies (All from Biolegend) for 45 min in the dark on ice. Cells were then washed and fixed with 4% paraformaldehyde in PBS (Alfa Aesar) for 15 min at room temperature and then permeabilized by washing twice with Intracellular Staining Permeabilization Wash Buffer (Biolegend) and stained with CD206-PE-Cy7 antibodies (Biolegend) for 45 min in the dark on ice for flow cytometric analysis using ACEA NovoCyte Flow Cytometer. A table of all antibody products and clones can be found in Supplemental Table 1.

### Transplant fluorescence imaging

Harvested lung digest cells were incubated in glass bottom, black walled 96-well plates. Following overnight adherence, cells were rinsed twice with PBS and then stained with CD45.1- Brilliant Violet 421™ (Biolegend) and with Cell Meter™ Live Cell TUNEL Apoptosis Assay Kit or Cell Navigator™ Lysosome Staining Kit (both from AAT Bioquest) according to manufacturer’s guidelines. Cells were imaged using BioTek Cytation 5 Multimode Imager. A table of all antibody products and clones can be found in Supplemental Table 1.

### Histology and immunohistochemistry

Tracheas were cannulated and filled with 1:1 OCT:PBS to fully inflate the lungs. Lungs were harvested, embedded in OCT, and flash frozen in liquid nitrogen. For histological analysis, lungs were cryosectioned into 7 µm sections. Sections were mounted to glass slides and stained using hematoxylin and eosin (H&E). For immunohistochemical staining, Ultra Streptavidin HRP Kit (Biolegend) was used to perform all staining steps according to manufacturer’s guidelines along with purified anti-mouse CD45.1 antibody (Biolegend) and sections were counterstained with hematoxylin. Stained sections were imaged using BioTek Cytation 5 Multimode Imager.

### Statistics

GraphPad Prism 9 (GraphPad Software Inc.) was used to perform statistical analyses. Numerical data are represented as mean ± standard deviation (SD) or standard error of the mean (SEM) as reported in the figure captions. Dunnett’s and Tukey’s multiple comparisons tests were used to generate *p*-values in ANOVA multiple comparisons, unless stated otherwise. Except for histological analyses, all results shown are representative of at least two independent experiments, with biological replicates reported in the figure captions.

## Results and discussion

### Nanoparticle pre-treatment enhances the survival of pulmonary macrophage transplant

**Fig. 1:**
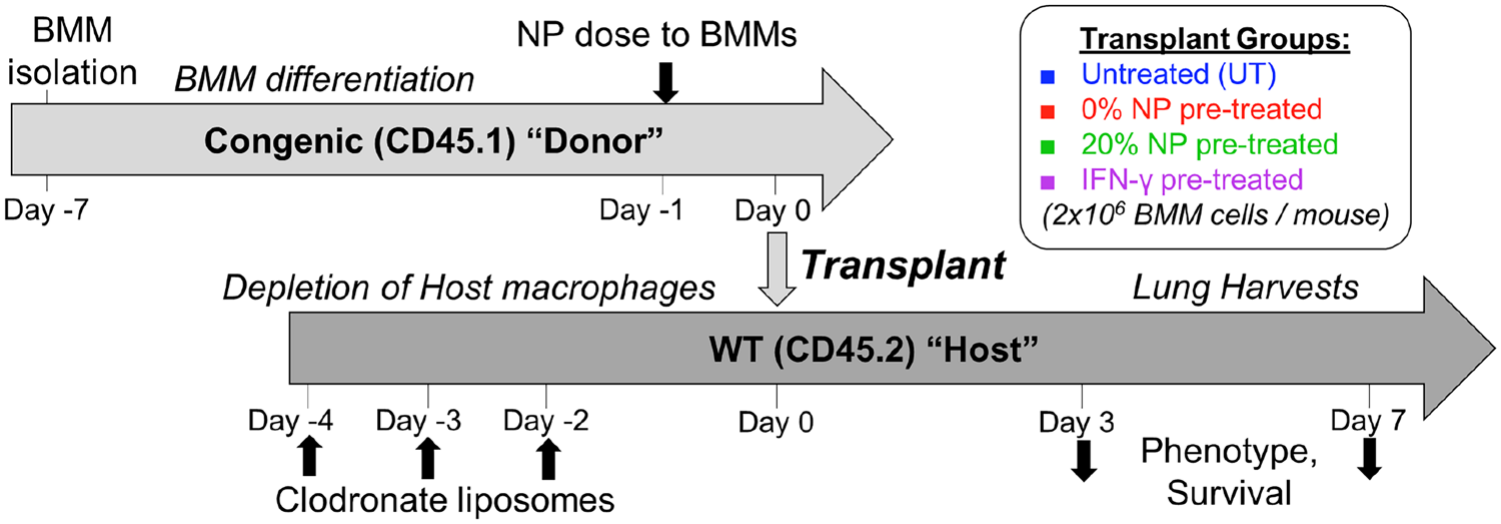
Pulmonary macrophage transplant (PMT) studies dosing schedule. BMMs were isolated from B6.SJL-*Ptprc*^*a*^* Pepc*^*b*^/BoyJ mice. In parallel, host C57BL/6 J mice were prepared for transplants by three daily orotracheal instillations of clodronate liposomes on Days − 4 through − 2. Transplants were performed on Day 0 and flow cytometric analysis on lung digests was performed on Days 3 and 7 for identification of CD45.1 + transplant survival and phenotypical analysis. Treatment groups are shown along with color legend for subsequent figures

We have previously demonstrated the ability of NPs to promote the ex vivo survival of primary macrophages through the activation of pro-survival lysosomal signaling and the suppression of pro-apoptotic caspase activity and DNA damage. Briefly, NP internalization, mainly through phagocytic routes, triggers the expression of late endosomal/lysosomal adaptor, MAPK and mTOR activator (LAMTOR) proteins, which are linked to survival, in addition to the upregulation of anti-apoptotic B cell lymphoma-2 (Bcl-2) proteins and the inhibition of executioner caspases 3 and 7 [15]. Based on our prior work demonstrating improved survival of ex vivo BMMs following NP treatment of various PEGDA chemistries [15, 16], we hypothesized that pre-treatment of pro-M1 NP formulations could increase the viability of PMT cells in vivo. To test our hypotheses regarding whether NP treatment and NP degradation rate impact the in vivo survival of PMT, ex vivo BMMs were dosed with 100 µg/ml of 0% NPs, 100 µg/ml 20% NPs, or left untreated for 24 h. Both NP formulations were ~ 300 nm in diameter with a negative zeta potential; characterization is shown in Supplemental Fig. S1. A fourth group of BMMs was treated with 25 ng/ml IFN-$\gamma$ for 24 h and used as a positive control for M1-like phenotype. To reduce the effect of host-transplant interactions and test our hypothesis of NP-induced enhanced cell survival, alveolar macrophage depletion was carried out using clodronate liposomes prior to PMT [20]. Supplemental Fig. S2 shows successful depletion of resident macrophages. BMMs from the four treatment groups were administered to mice (2.0 × 10^6^ cells per mouse) according to the dosing schedule in Fig. 1. At the Day 3 and Day 7 timepoints, flow cytometric analysis was used to identify the counts of live transplant cells. Representative flow cytometry gating for identifying transplant cells is shown in Supplemental Fig. S3. Low transplant viability in this PMT model has been observed by others [17], indicating key challenges in transplant retention in healthy wild type mice.

**Fig. 2:**
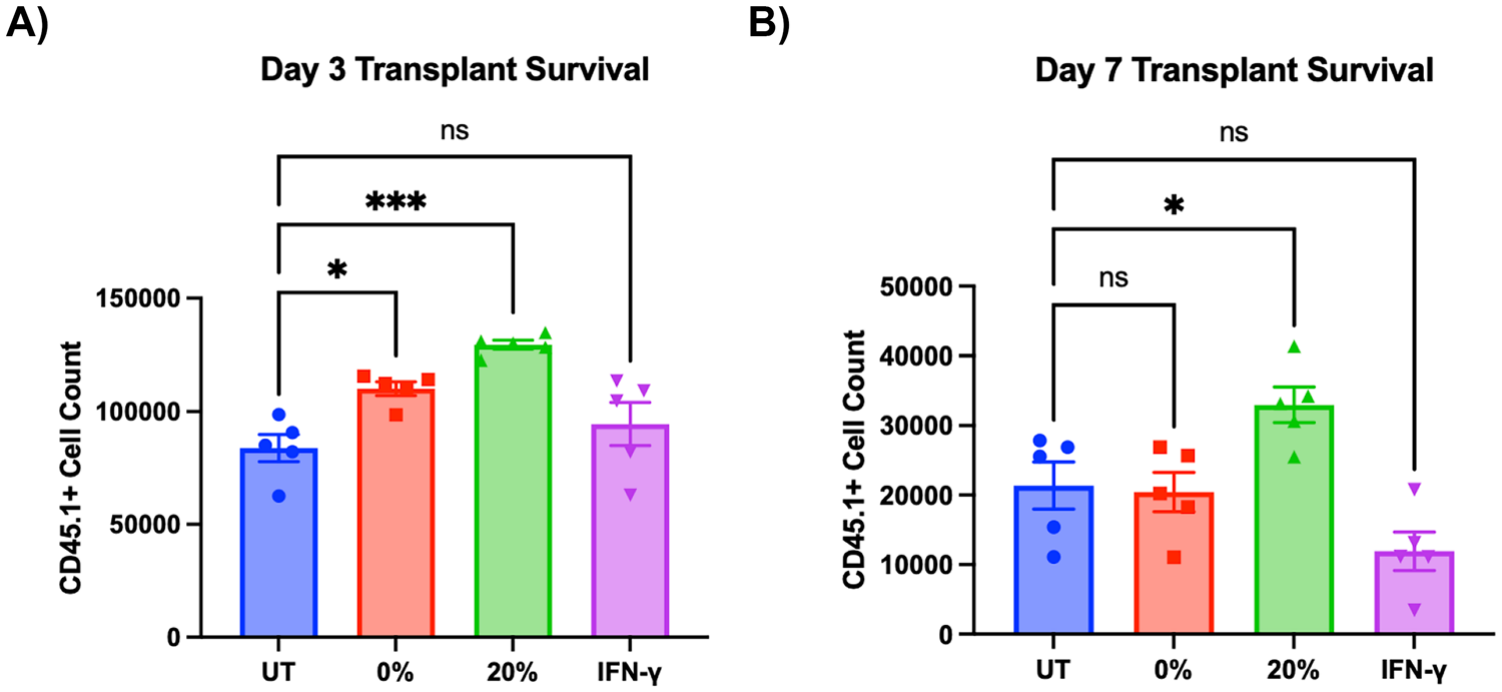
Survival of CD45.1 + BMMs on **A** Day 3 and **B** Day 7 following PMT in whole lung digests. *p < 0.05 and ***p < 0.001; ns is not significant (compared to untreated transplant, UT) using Dunnett’s multiple comparisons test (one-way ANOVA) (*N* = 5 mice; representative results from duplicate experiments). Bars represent the mean and error bars represent SEM

As shown in Fig. 2, transplant of BMMs pre-treated with either the 0% or 20% NP formulation resulted in statistically significantly higher survival of transplanted cells than the untreated (UT) cells 3 days following PMT (*p* < 0.05 and *p* < 0.001 for 0% and 20% NPs, respectively using Dunnett’s multiple comparisons tests as part of a one-way ANOVA). This is in contrast to BMMs pre-stimulated with IFN-γ, which did not result in any statistically significant changes to cell survival 3 days after PMT (*p* > 0.05 using Dunnett’s multiple comparisons tests as part of a one-way ANOVA). Of the two formulations, the rapidly degrading pro-M1 20% NPs resulted in the highest PMT survival levels across all the tested treatments at Day 3, where the 0% NPs resulted in 31% higher cell counts and the 20% NPs caused a 54% increase in transplant survival. This trend was recapitulated on Day 7; while overall numbers of remaining transplanted cells had decreased in all conditions, only the BMMs pre-treated with the 20% NPs showed increased survival over the UT cells, with 54% higher retention in transplanted macrophage counts (*p* < 0.05 using Dunnett’s multiple comparisons tests as part of a one-way ANOVA). At this timepoint, the 0% NP pre-treatment did not provide any increased PMT cell survival, while pre-treatment with IFN-γ resulted in a 44% lower survival compared to untreated transplants. Results from these PMT studies mirror prior ex vivo survival results of BMMs dosed with NPs of varying HS-PEG-SH content, where 20% NPs resulted in the greatest ex vivo survival compared to the lower HS-PEG-SH content formulations and untreated cells [16]. The results shown in Fig. 2 support our hypothesis that NP formulation plays a major role in regulating the survival of the phagocytosing cell, which appears to not only be applicable to ex vivo settings but also in in vivo transplants. These results are the first to report a significant benefit of macrophage pre-treatment in enhancing phagocytic cell survival, and while the overall improvement is relatively modest, these important discoveries open the door to the leveraging this strategy towards continual improvement of macrophage-based cell therapies via NP pre-treatment.

**Fig. 3:**
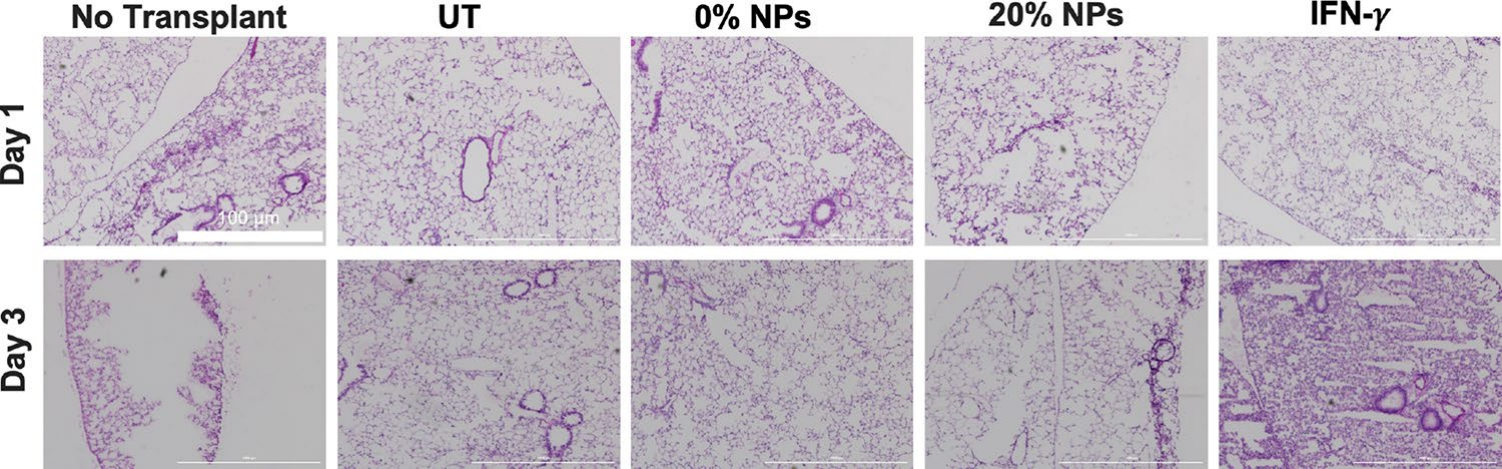
H&E histological analysis (4 × magnification) of lungs at Days 1 and 3 from mice receiving no transplant, untreated transplant, 0% NP-treated transplant, 20% NP-treated transplant, and IFN-$\gamma$-treated transplant. Scale bar 100 µm

Histological analysis of H&E-stained sections (Fig. 3) revealed that lung sections of 0% and 20% NP-treated PMTs were visually indistinguishable from those from mice receiving no transplants (negative control), with respect to cellularity and the presence of infiltrating inflammatory cells, at both Day 1 and Day 3 post-transplant. This was also the case for mice receiving untreated transplant cells. Unsurprisingly, PMTs stimulated with IFN-$\gamma$ caused notable recruitment of infiltrating inflammatory cells at Day 3 post-transplant, which may point to potential rejection of IFN-$\gamma$ transplants and potentially disadvantageous effects of this treatment group. The low levels of airway inflammation reflected in mice receiving NP-treated PMTs demonstrate the host receptiveness of NP-treated PMTs with no signs of overt inflammation, which is in line with previous reports of PMT of naïve BMMs in mouse lungs [17]. Interestingly, these previous reports show that the transplanted BMMs adopt a phenotype close to that of host alveolar macrophages and show a conversion from CD11b^Hi^Siglec-F^Low^ to CD11b^Low^Siglec-F^Hi^ [17]. These observations point to the potential adaptability of the PMT and long-term tolerability by the host lung immune environment that would need to be confirmed in follow-on studies.

**Fig. 4:**
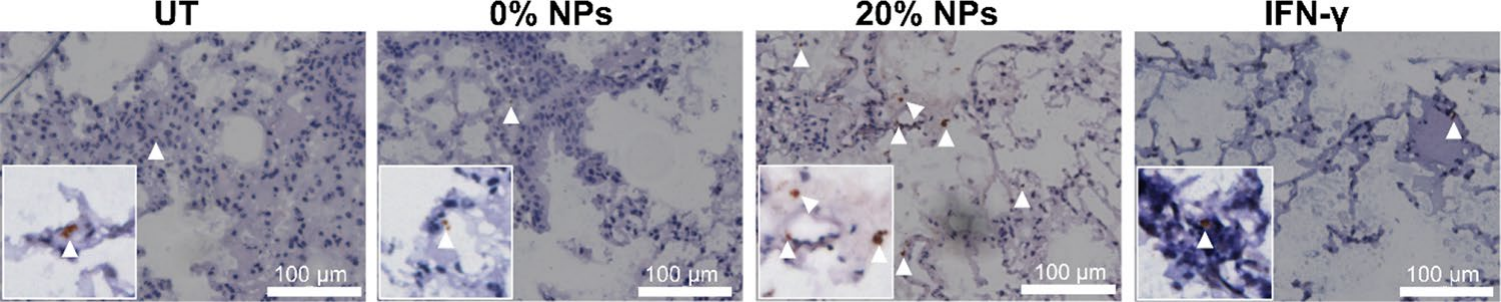
Detection of CD45.1 + transplant cells in lung sections with immunohistochemistry at Day 3 from mice receiving untreated transplant, 0% NP-treated transplant, 20% NP-treated transplant, and IFN-$\gamma$-treated transplant (20 × magnification). Scale bar 100 µm. Insets show 40 × magnification demonstrating transplanted CD45.1 cells in each group. Arrows indicate the presence of CD45.1 + transplant cells

To confirm NP-induced survival patterns as seen in Fig. 2, immunohistochemical analysis of lung sections and staining for CD45.1 + transplants was carried out (Fig. 4). As seen from brown stained cells in images from immunohistochemical staining (indicated by white arrows), the frequency of CD45.1 + transplant cells was highest in 20% NP-treated transplants, which corresponds to the results from flow cytometric detection of transplant cells. Transplants treated with 0% NPs or IFN-$\gamma$ showed similar numbers of CD45.1 + events compared to untreated transplants. However, it is noteworthy to mention the relatively low raw cell number in these thin lung sections used for histological or immunohistochemical analysis. Nevertheless, visual results from lung sections indicate that 20% NPs show higher abundance of macrophage transplants in lung tissue.

### Nanoparticle pre-treatment to transplanted macrophages promotes lysosomal engagement and anti-apoptotic activity

**Fig. 5:**
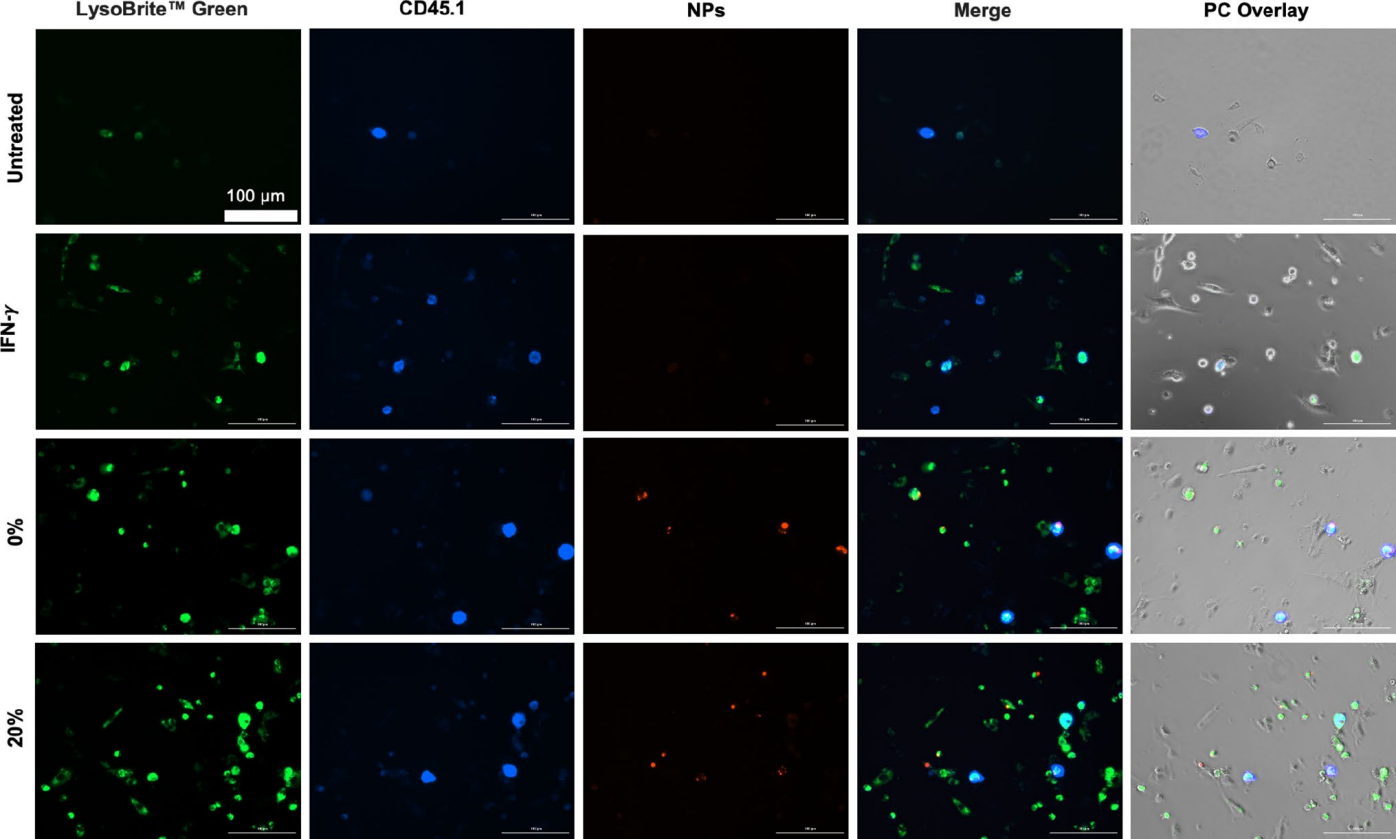
Representative lysosomal tracking and imaging at 20 × magnification with LysoBrite™ Green of lung digest cells on Day 3 post-transplant of BMMs treated with 100 µg/ml of 0% and 20% NPs, 25 ng/ml IFN-$\gamma$, or untreated BMMs. Scale bar 100 µm. Phase contrast (PC). Images are representative of three biological replicates; results representative of duplicate experiments

To investigate the effect of degradable NP pre-dosing on intracellular processing in PMT cells, lung digest cells obtained on Day 3 post-transplant were stained with LysoBrite™ Green and imaged to detect changes in lysosomal activity. Figure 5 shows high-intensity LysoBrite™ Green activity in all NP-treated PMT cells compared to their untreated counterparts, indicating NP trafficking to late lysosomal compartments. Furthermore, the LysoBrite™ Green signal was the most abundant in 20% NP-treated PMT, which points to increased lysosomal activity in PMTs dosed with rapidly degradable NPs compared to slowly degrading NPs or untreated PMTs. Lysosomal activity reflects previous ex vivo results in BMMs treated with degradable formulations [16] that is directly linked to cell survival mechanisms [15] and are generally expected because of target degradation in intracellular compartments including in the phagolysosome, which forms as a result of fusion of the phagosomal and lysosomal compartments containing internalized NPs [23].

**Fig. 6:**
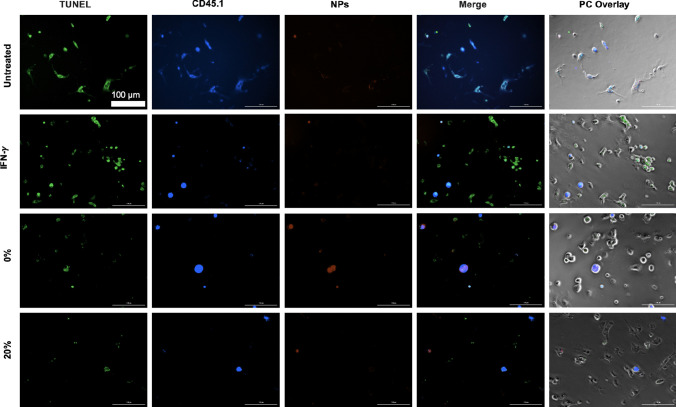
Representative TUNEL apoptosis imaging analysis at 20 × magnification of lung digest cells on Day 3 post-transplant of BMMs treated with 100 µg/ml of 0% and 20% NPs, 25 ng/ml IFN-$\gamma$, or untreated BMMs. Scale bar 100 µm. Phase contrast (PC). Images are representative of three biological replicates; results representative of duplicate experiments

TUNEL imaging analysis revealed notably lower DNA damage (late stage of apoptosis) in NP-treated PMT cells compared to untreated PMT counterparts on Day 3 post-transplant (Fig. 6). Twenty percent NP-treated PMT showed lowest fluorescent TUNEL abundance, indicating lower late apoptosis characterized by DNA damage in PMTs with NP pre-treatment. Combined with results presented in Fig. 5, these images suggest that NP pre-treatment enhances anti-apoptotic survival of PMT cells, avoiding apoptosis through prevention of DNA damage and enhanced lysosomal signaling. We have previously shown that NP internalization stimulates the expression of late endosomal/lysosomal adaptor, MAPK and mTOR activator (LAMTOR) genes and proteins [15], which have been linked to cell survival [24, 25]. The enhanced lysosomal activity may potentially trigger increased expression of lysosomal signaling proteins, which have been reported to contribute to cell survival. Administration of biodegradable NPs with acidic byproducts have been shown to restore lysosomal acidity and degradative capacity [26, 27], which may further contribute to cell stimulation. Combined with supporting studies of pro-survival signaling centered in the lysosome [28, 29], this prior work highlights an untapped opportunity for intelligently designed NP platforms to further modulate this response. Moreover, these results demonstrate that enhanced lysosomal activity is critical in regulating cell viability in vivo and enhances efficacy of transplant viability within a complex in vivo microenvironment. Collectively, the results presented in Figs. 2, 3, 4, 5, and 6 demonstrate that pre-treatment with NPs affords an advantageous, cost-effective, and well-tolerated route to increasing the survival of macrophage therapies in PMT by engaging lysosomal pro-survival signals that persist in vivo following transplant.

### Nanoparticle pre-treatment preserves the phenotype of PMT

While the prior figures demonstrate that NP pre-treatment can enhance the overall survival of PMT cells in vivo, we next sought to investigate the effect of NP formulation on PMT phenotype retention following transplant. BMMs were analyzed for activation markers following NP pre-treatment, both before and after PMT.

**Fig. 7:**
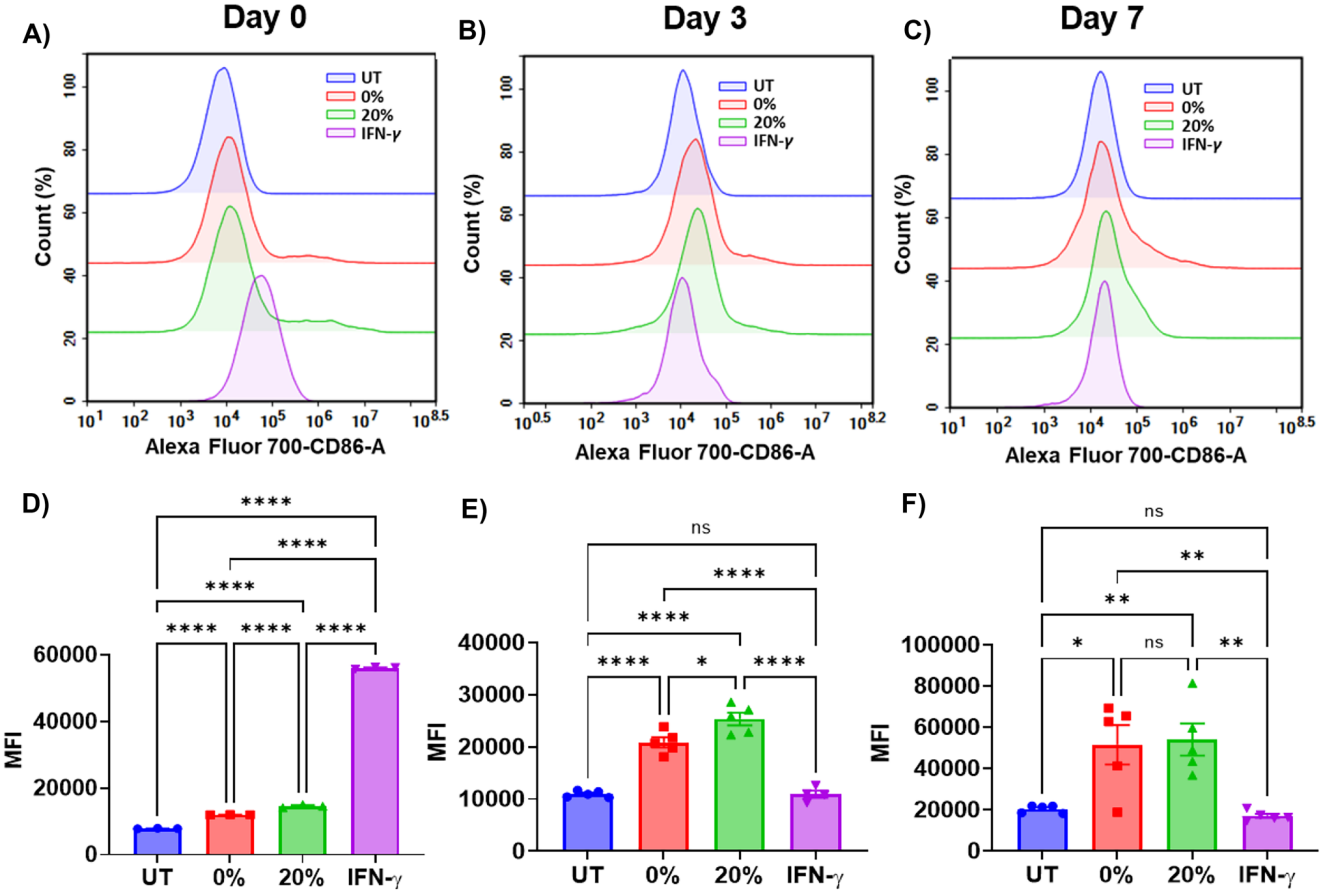
Expression of representative CD86 activation marker of CD45.1 + BMMs treated with 100 µg/ml of 0% and 20% NPs, 25 ng/ml IFN-$\gamma$, or untreated BMMs for 24 h. **A**, **D** Pre-transplant (Day 0). **B**, **E** Day 3 PMT. **C**, **F** Day 7 PMT. Top panel: Representative flow cytometric histograms of CD86 expression of CD45.1 + BMMs. Bottom panel: CD86 median fluorescence intensity of CD45.1 + BMMs. *p < 0.05, **p < 0.01, and ****p < 0.0001; ns is not significant using Tukey’s multiple comparisons tests as part of a one-way ANOVA (*N* = 3–5 mice; results representative of duplicate experiments). Error bars represent SEM

**Fig. 8:**
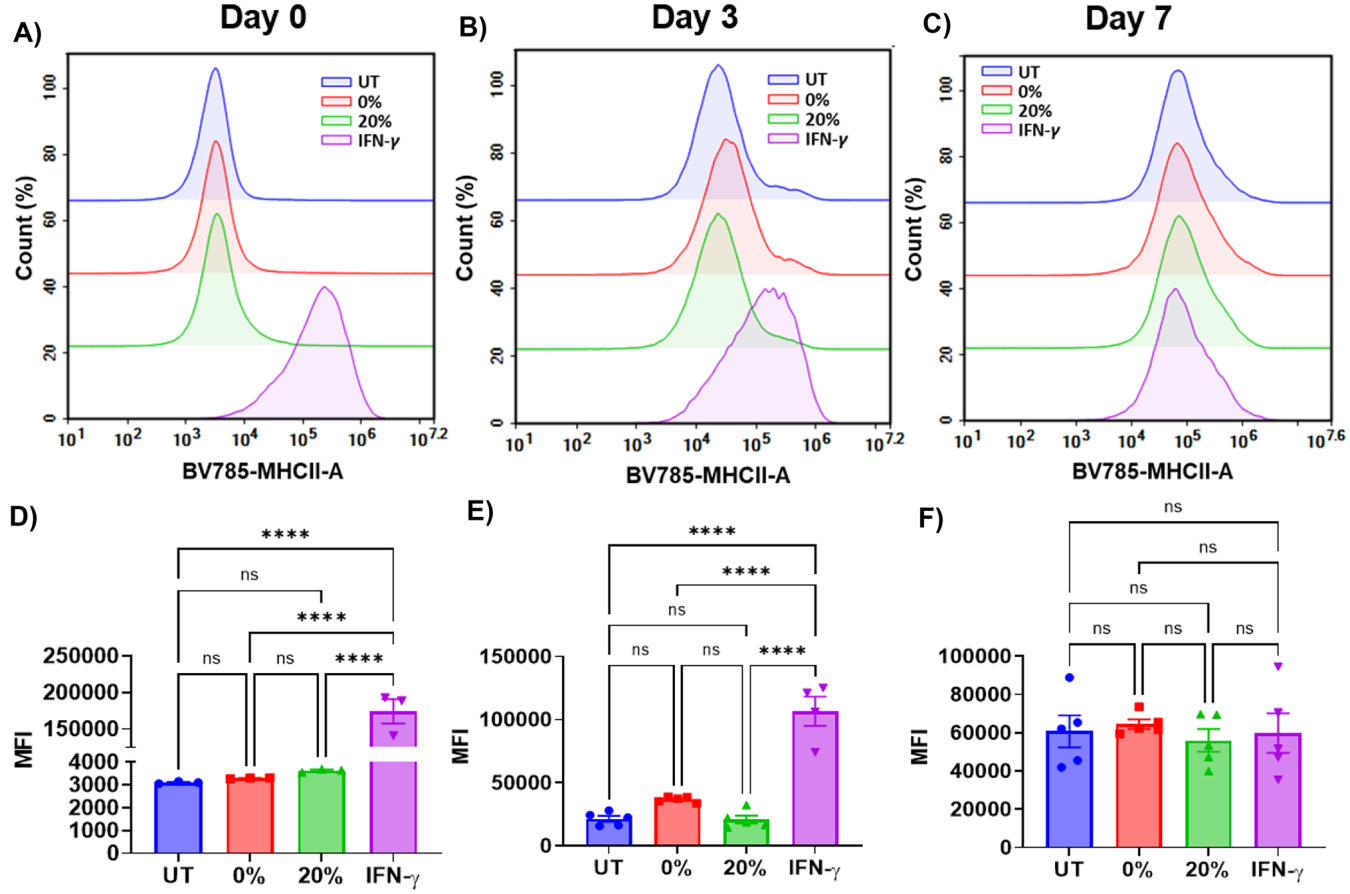
Expression of representative MHCII activation marker of CD45.1 + BMMs treated with 100 µg/ml of 0% and 20% NPs, 25 ng/ml IFN-$\gamma$, or untreated BMMs for 24 h. **A**, **D** Pre-transplant (Day 0). **B**, **E** Day 3 PMT. **C**, **F** Day 7 PMT. Top panel: Representative flow cytometric histograms of MHCII expression of CD45.1 + BMMs. Bottom panel: MHCII median fluorescence intensity of CD45.1 + BMMs. ****p < 0.0001; ns is not significant using Tukey’s multiple comparisons tests as part of a one-way ANOVA (*N* = 3–5 mice; results representative of duplicate experiments). Error bars represent SEM

Prior to PMT (Fig. 7A, D), CD86 M1-like activation marker showed significantly higher expression in 0% and 20% NP-treated BMMs compared to untreated cells. NPs containing HS-PEG-SH caused higher levels of activation compared to 0% NPs, confirming previous results that the 20% degradable NPs can polarize macrophages towards an M1-like state [16]. Furthermore, analysis of NP + populations showed further enhancement of the expression of CD86 costimulatory marker (Supplemental Fig. S4). Unsurprisingly, IFN-$\gamma$ treatment caused potent M1-like polarization characterized by drastically higher levels of CD86 and MHCII expression compared to all the other treatment groups. However, unlike IFN-$\gamma$, MHCII expression was not statistically significantly upregulated (*p* > 0.05 using Tukey’s multiple comparisons tests as part of a one-way ANOVA) (Fig. 8A, D). CD206 expression, as a representative M2 marker [14], was not statistically significantly different in 0% and 20% NP-treated BMMs compared to untreated cells (*p* > 0.05 using Tukey’s multiple comparisons tests as part of a one-way ANOVA); however, IFN-$\gamma$ treatment resulted in significantly higher CD206 expression compared to the other treatments (Supplemental Fig. S5). Thus, all treatment conditions yielded variable levels of M1-like activation prior to PMT, with likely some degree of heterogeneity within the transplanted population.

Following PMT, NP-treated BMM transplant showed significantly higher levels of CD86 costimulatory molecule compared to the untreated transplant counterpart on both Day 3 (Fig. 7B, E) and Day 7 (Fig. 7C, F) (*p* < 0.05 using Tukey’s multiple comparisons tests as part of a one-way ANOVA). CD86 expression was 90% and 132% higher than that of untreated transplants in 0% and 20% NP-treated transplants, respectively, on Day 3 and 20% NPs caused significantly higher CD86 expression compared to 0% NPs (*p* < 0.05 using Tukey’s multiple comparisons tests as part of a one-way ANOVA), indicating the stimulatory effect of rapidly degrading NPs compared to their slowly degrading counterparts. At the Day 7 timepoint, CD86 expression for 0% and 20% NP-treated transplants was 153% and 165% higher than that of untreated transplant, respectively. Interestingly, both 0% and 20% NP-treated transplant caused significantly higher CD86 expression compared to IFN-$\gamma$-treated transplant on both timepoints (*p* < 0.0001 using Tukey’s multiple comparisons tests as part of a one-way ANOVA), which indicates that NP treatment has the potential to cause potent and long-lasting phenotypical changes to transplanted macrophages when compared to commonly used soluble stimuli (e.g., IFN-$\gamma$), where IFN-$\gamma$-treated PMT resulted in statistically insignificantly different levels of CD86 expression compared to the untreated transplant (*p* > 0.05 using Tukey’s multiple comparisons tests as part of a one-way ANOVA). Similar patterns of NP-induced CD86 stimulation are observed in analysis of NP + subpopulations (Supplemental Fig. S6B), where higher CD86 MFI levels in CD45.1 + /NP + cells were observed compared to those of the total population. Overall, post-transplant CD86 patterns for 0% and 20% NP-treated conditions followed those of pre-transplant cells, which was not the case for IFN-$\gamma$, where sharp decay of CD86 expression was observed post-transplant as early as Day 3, resulting in statistically insignificant levels compared to untreated transplants.

Contrary to patterns observed with CD86 costimulatory molecule, significant changes to MHCII expression compared to untreated PMT were only observed in transplanted cells treated with IFN-$\gamma$ at the Day 3 timepoint (*p* < 0.0001 using Tukey’s multiple comparisons tests as part of a one-way ANOVA) and not with the NP-treated conditions (Fig. 8E, F), although MHCII expression was 72% higher in 0% NP-treated transplants compared to their untreated counterparts. Similar to CD86, high MHCII expression in IFN-$\gamma$-treated transplants was only present until the Day 3 timepoint and seemed to decay by Day 7. Results of insignificant MHCII expression following degradable NP treatment are in disagreement with previous results showing potent M1-like stimulation up to 3 days following treatment with degradable NP formulations [16], and may point to influence of local in vivo stimuli influencing MHCII expression in situ. Higher MHCII expression is expected with macrophages treated with potent M1-polarizing stimuli like IFN-$\gamma$. Achieving more potent MHCII expression levels may be possible by incorporating soluble IFN-$\gamma$ or other potent M1 stimuli in combination with 0% and 20% NPs and pre-treating to cells prior to transplants. In addition, an alternative approach would entail encapsulation of soluble stimuli in the NP formulations for a sustained release following internalization by transplant cells.

Similar to pre-transplant results, CD206 expression was not statistically significantly different in 0% and 20% NP-treated BMMs compared to their untreated counterparts (*p* > 0.05 using Tukey’s multiple comparisons tests as part of a one-way ANOVA) (Supplemental Fig. S7). However, rapidly degrading 20% NPs showed statistically significant downregulation of CD206 expression compared to 0% NP- and IFN-$\gamma$-treated transplants. These patterns were held in analysis of NP + populations, which revealed lowest CD206 expression in 20% NP-treated BMMs (Supplemental Fig. S7). Similar to pre-transplant, CD206 expression results were surprising given previous results with degradable HS-PEG-SH formulations [16], which indicates likely differences and potential transience in CD206 expression in different environments.

Collectively, our results demonstrate that pre-treatment with NP formulations of controlled chemistry can be leveraged to tune the extent and duration of macrophage phenotype retention, even within a complex in vivo environment. Moreover, our results demonstrate that this phenotype retention can be obtained without additional macromolecule cargo and can be tuned relative to the degradation kinetics of the NP. We hypothesize that this pre-treatment strategy can provide significant inexpensive opportunities for cellular therapies and promote the adoption of CAR M approaches, by overcoming existing challenges in the translational workflows [9, 30]. Notably, phenotype retention is a challenge for macrophage cellular therapeutics within the tumor immune microenvironment, which drive macrophages towards an immunosuppressive phenotype [31]. Therefore, promoting a sustained M1 phenotype is especially important in developing macrophage-based cancer immunotherapies [32]. From the results in Fig. 7 and previous ex vivo work [16], we demonstrate the ability to retain a prolonged M1-like response in macrophages treated with degradable NPs, which has shown an advantage over IFN-$\gamma$ pre-treated macrophages. While our work has yet to address whether the pro-M1 phenotype can persist in a strong M2-polarizing environment, NP-induced phenotype retention may be combined with soluble M1-inducing stimuli including IFN-$\gamma$ in future studies and in potential clinical translation to elicit more robust and amplified M1-like response. Along with recent efforts including CAR constructs [7] and macrophage backpacks [33], our findings demonstrate an additional avenue by which macrophage-based cell therapies can be improved. Furthermore, our overall results demonstrating enhanced macrophage transplant efficiency in a PMT model may be advantageous for improving the clinical translation of life-saving cellular transplant approaches for diseases such as pulmonary alveolar proteinosis (hPAP) [17], where an M1-phenotye retention is less advantageous. In the context of clinical translation of pulmonary macrophage transplantation therapies, several limitations remain outstanding. As transplanted macrophages are terminally differentiated and are unable to proliferate in vivo without the addition of high amounts of growth factors, there is a considerable drop in the total number of transplanted macrophages, which is also observed in the drop between Day 3 and Day 7 (Fig. 2). This is expected, as macrophages have been shown to undergo rapid apoptosis when growth factors, e.g., M-CSF, are depleted [34]. Repeated dosing may be required to achieve therapeutic efficacy as this approach is applied to functional cell therapies, such as CAR M approaches [9]. While we provide a NP-based approach to enhance the persistence of transplanted macrophages, alternative mitigation strategies can be considered, including the incorporation of cell proliferation stimuli and an optimized dosing schedule, to ensure robust therapeutic efficacy. Furthermore, the explored route of administration of transplanted cells in our study is through the pulmonary route, which depending on the method and frequency of administration could introduce additional complications in clinical translation, which must be evaluated depending on the disease in question. While the pulmonary route is an attractive target for delivery in achieving localized responses in respiratory conditions, other administration routes and subsequent tissue localization are likely more appropriate for many CAR M applications [6] and should be investigated to further advance the understanding of in vivo fate of macrophage transplants. In addition, the proposed in vivo model for PMT in this study could be further investigated to optimize transplantation parameters and facilitate clinical translation, including consideration of alternatives to clodronate liposomes for ablating host macrophage populations and evaluating how the host-transplant interactions affect therapeutic efficacy.

## Conclusions

The results presented in this work show the ability of NP pre-treatment to enhance the survival of macrophage transplants. NPs pre-dosed to BMMs caused improved survival upon pulmonary transplant compared to untreated BMMs. Furthermore, rapidly degradable pro-M1 PEGDA-based NP formulations were superior to their slowly degrading counterparts in driving PMT survival and retaining an M1 phenotype. We showed that degradable NP dosing to macrophage transplant causes prolonged M1-like stimulation in vivo that out-performed a potent cytokine stimulus, highlighting a potential benefit in using this platform as a cell-based immune engineering strategy for extended phenotype influence over a phagocytic cell therapy. These findings represent proof-of-concept demonstrations showing potential employment of NP strategies in macrophage-based cell therapies.

### Supplementary Information

Below is the link to the electronic supplementary material.

Supplementary file1 (PDF 1677 kb)

## Data Availability

The datasets generated during and/or analyzed during the current study are available from the corresponding author on reasonable request.
