# Supplementary material for: Nanoparticle pre-treatment for enhancing the survival and activation of pulmonary macrophage transplant
Source: Drug Deliv Transl Res. 2023 Mar 14;13(7):1955–66. doi: 10.1007/s13346-023-01319-6 (PMC10238309; doi:10.1007/s13346-023-01319-6)
Supplement: Supplementary file 1 — Supplementary file1 (PDF 1677 kb) [file 13346_2023_1319_MOESM1_ESM.pdf]

Supporting Information for:

Nanoparticle Pre-Treatment for Enhancing the Survival and Activation of Pulmonary Macrophage Transplant

Bader M. Jarai[†], Kartik Bomb[†], Catherine A. Fromen, PhD[†, *]

†Department of Chemical and Biomolecular Engineering, University of Delaware, Newark, DE 19716

*corresponding author.
cfromen@udel.edu
150 Academy St.
Newark, DE 19716
(302) 831-3649

Table S1: Product information for antibodies used in flow cytometry, immunostaining, and IHC.

Antibody	Supplier	Product #	Clone	Isotype
Anti-mouse CD16/32	Biolegend	156604	S17011E	Rat IgG2b, κ
Anti-mouse CD45.1-Pacific Blue	Biolegend	110722	A20	Mouse (A.SW) IgG2a, κ
Anti-mouse CD86-AlexaFluor700	Biolegend	105024	GL-1	Rat IgG2a, κ
Anti-mouse I-A/I-E-Brilliant Violet 785™	Biolegend	107645	M5/114.15.2	Rat IgG2b, κ
Anti-mouse CD206-PE-Cy7	Biolegend	141720	C068C2	Rat IgG2a, κ
Anti-mouse CD45.1-Brilliant Violet 421™	Biolegend	110732	A20	Mouse (A.SW) IgG2a, κ
Anti-mouse Purified CD45.1	Biolegend	110702	A20	Mouse (A.SW) IgG2a, κ
Anti-mouse Siglec-F- APC-Cy7	BD Biosciences	565527	E50-2440	Rat IgG2a, κ

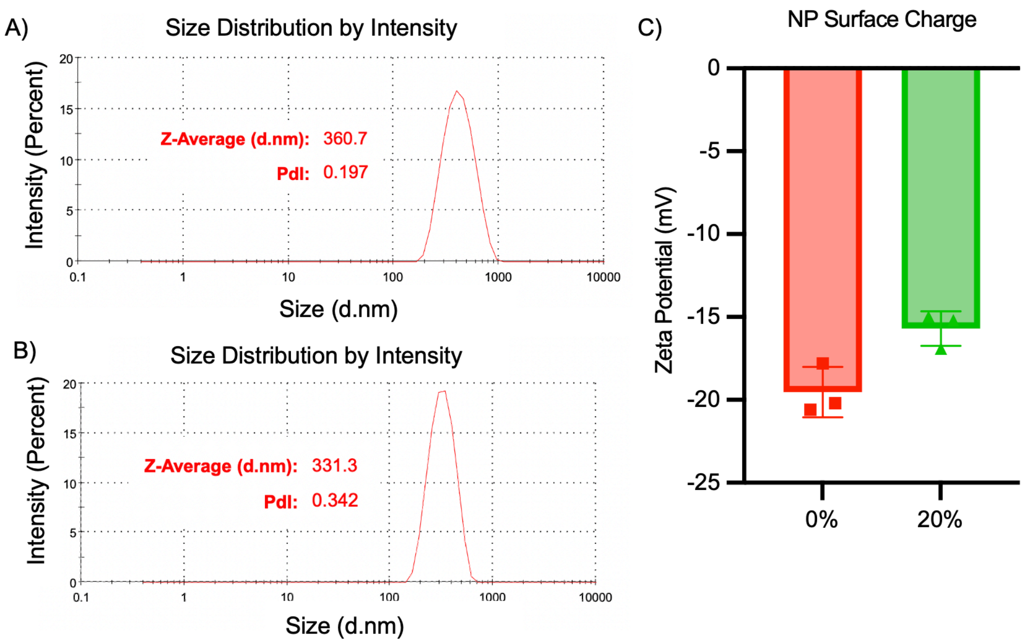

Figure S1: Nanoparticle characterization of PEGDA NPs 0% and 20% used in this study.

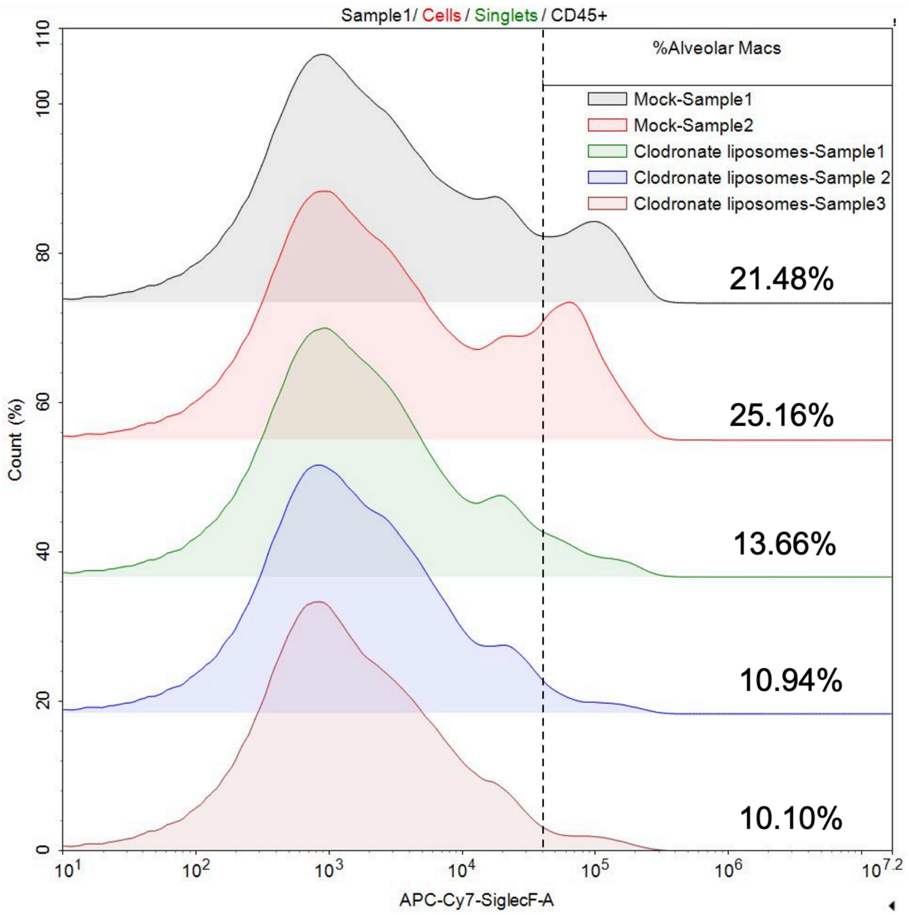

Figure S2: Histograms of Siglec-F alveolar macrophage expression in CD45+ populations of lung digests following 3 doses of clodronate liposomes measured on Day 0 (Figure 1). Mock-Sample 1 and Mock-Sample 2 represent lung digests of mice receiving PBS while Clodronate liposomes-Sample 1, 2, and 3 represent lung digests of mice receiving clodronate liposomes.

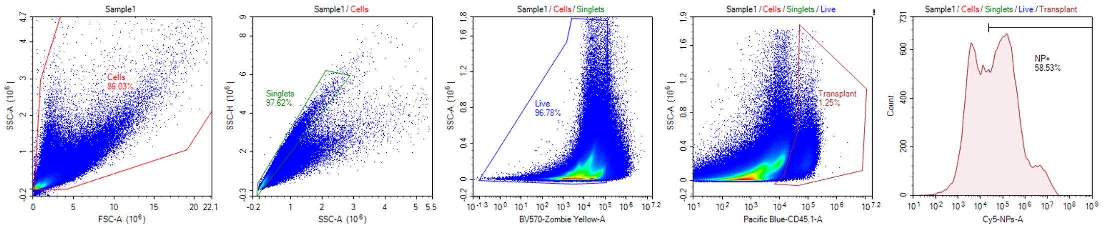

Figure S3: Sample gating of flow cytometric analysis of PMT. Flow cytometric analysis was performed on samples from whole lung digests: Events from SSC/FSC plot of samples of whole lung digests were gated to exclude debris and then gated to restrict analysis to single cell populations. Singlets were probed for Zombie Yellow expression and Zombie Yellow⁻ (Live) cells were investigated for CD45.1+ (Transplant) populations. Histograms were used to monitor NP+ populations and fluorescence of activation markers.

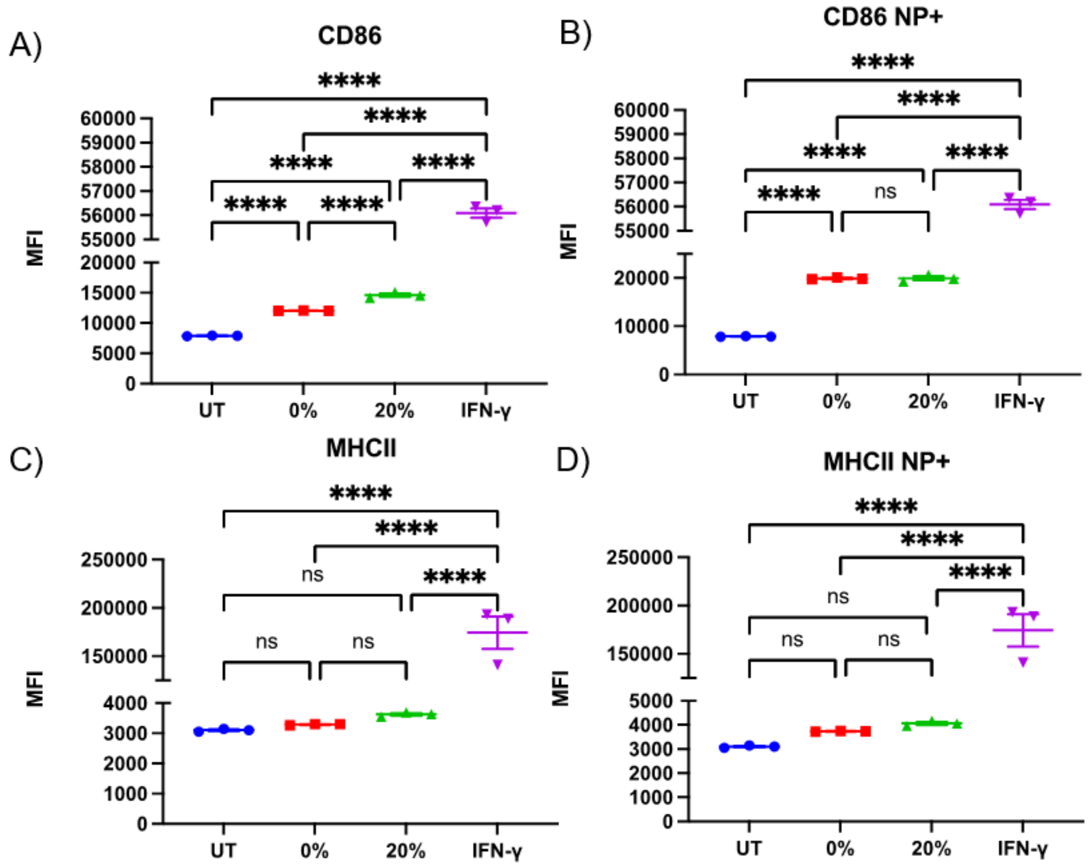

Figure S4: Pre-transplant expression of representative activation markers of CD45.1+ BMMs treated with 100 µg/ml of 0% and 20% NPs, 25 ng/ml IFN-γ, or untreated BMMs measured at Day 0. A) Total population CD86 expression B) NP+ CD86 expression C) Total population MHCII expression D) NP+ MHCII expression.

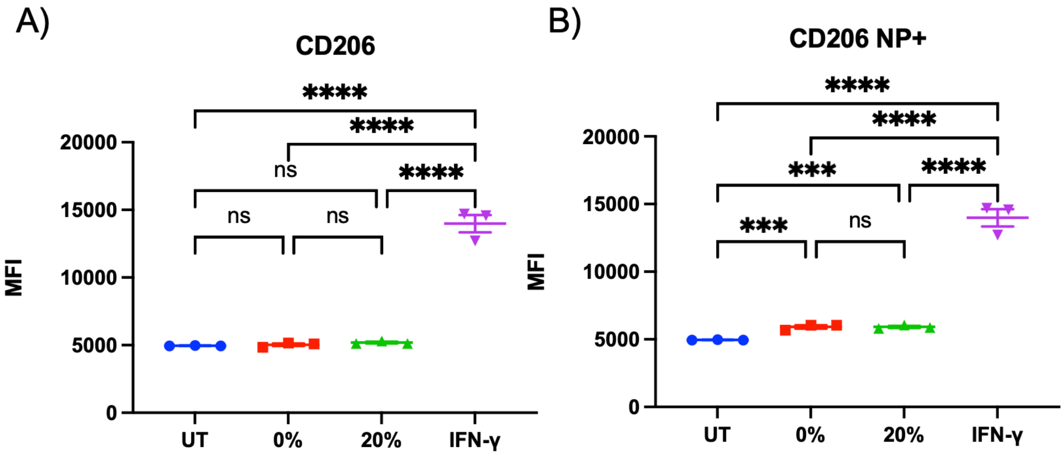

Figure S5: Pre-transplant CD206 expression of representative activation markers of CD45.1+ BMMs treated with 100 µg/ml of 0% and 20% NPs, 25 ng/ml IFN-γ, or untreated BMMs measured at Day 0. A) CD206 expression in total CD45.1+ population. B) CD206 expression in CD45.1+/NP+ population. *p<0.05, **p<0.01, ***p<0.001, ****p<0.0001, ns is not significant using Tukey's multiple comparisons tests as part of a one-way ANOVA (N=5). Error bars represent SEM.

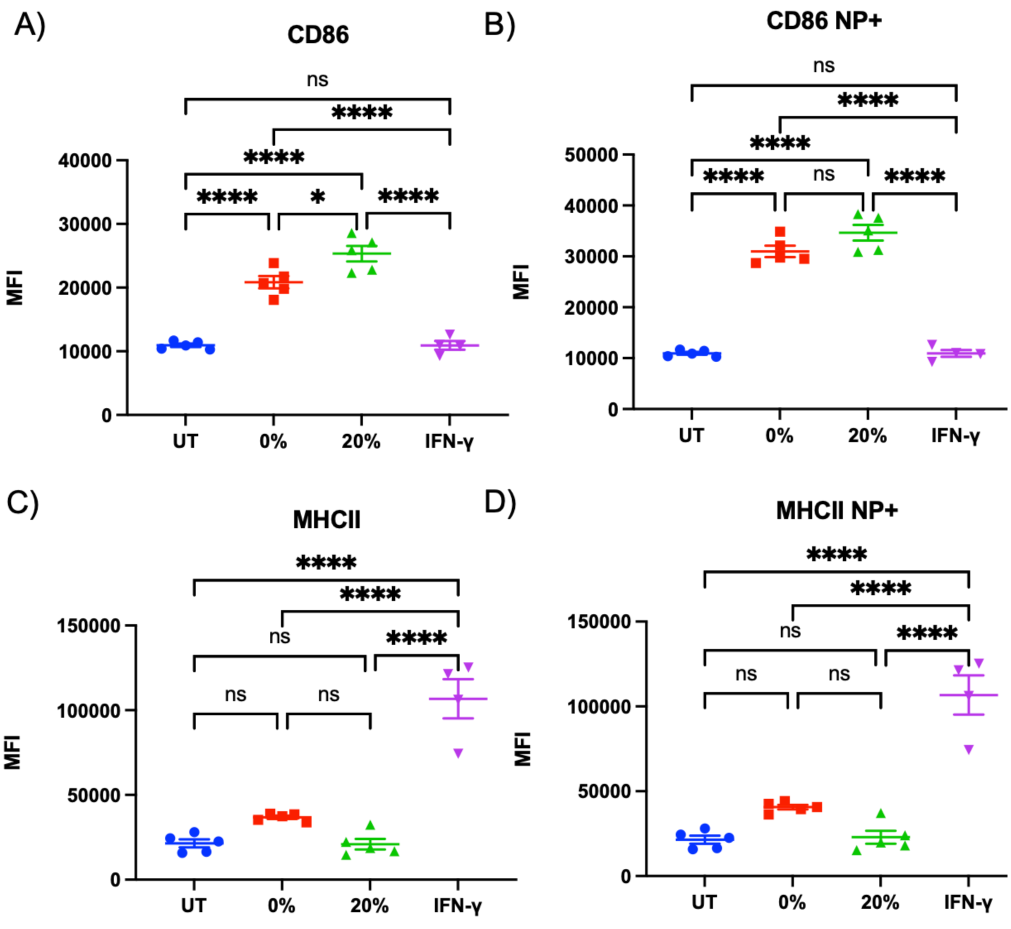

Figure S6: PMT expression of representative activation markers of CD45.1+ BMMs treated with 100 µg/ml of 0% and 20% NPs, 25 ng/ml IFN-γ, or untreated BMMs measured at Day 3. A) CD86 expression in CD45.1 populations. B) CD86 expression in CD45.1+/NP+ population. C) MHCII expression in CD45.1 population. D) MHCII expression in CD45.1+/NP+ population. *$p<0.05$, **$p<0.01$, ***$p<0.001$, ****$p<0.0001$, ns is not significant using Tukey's multiple comparisons tests as part of a one-way ANOVA (N=5). Error bars represent SEM.

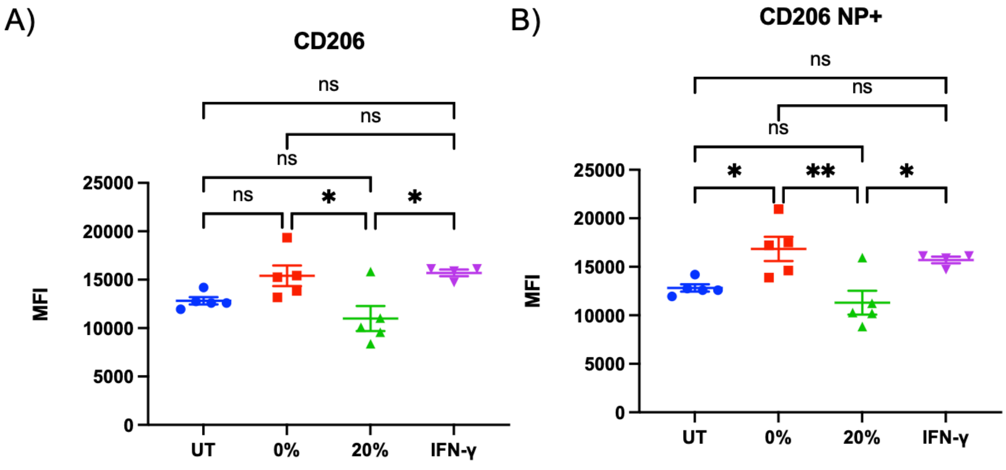

Figure S7: PMT expression of CD206 marker of CD45.1+ BMMs treated with 100 µg/ml of 0% and 20% NPs, 25 ng/ml IFN-γ, or untreated BMMs measured at Day 3. A) CD206 expression in CD45.1 population. B) CD206 expression in CD45.1+/NP+ population. *p<0.05, **p<0.01, ***p<0.001, ****p<0.0001, ns is not significant using Tukey's multiple comparisons tests as part of a one-way ANOVA (N=5). Error bars represent SEM.
